# Treatment of a Tumor with Iodic Acid

**Published:** 1895-10

**Authors:** Wilfried Lellmann

**Affiliations:** New York City


### REPORTS OF CASES.

### TREATMENT OF A TUMOR WITH IODIC ACID.

BY DR. WILFRIED LELLMANN,
NEW YORK CITY.

DURING last summer I was called to examine a mare having a tumor of the mammary gland. The owner of the animal told me that he had noticed the tumor long ago, and that it had grown gradually to the present size.

The result of my examination was about the following: An old white mare, about thirteen years of age, about sixteen hands high, weighing about 1400 pounds. The mare was in quite a good condition, and a thorough internal examination could not define anything abnormal. By examining the mammary gland I found the tumor in the left half of the gland, the right half being normal. The tumor was about the size of a child's head, of an oblong shape. Palpation proved the tumor to be of hard consistency, broad at its base, and becoming wedge-shaped toward the pubis. The surface of the tumor was even, and the skin adherent to the tumor around the left mammary process, which showed a distinct retraction, being considerably shorter than the right process. The supra-mammary glands were not swollen. The tumor seemed to be absolutely painless.

*Diagnosis*. "Adeno-fibroma mammæ." After the examination I told the owner that I thought it was not a malignant tumor, but if he wished to have the tumor removed I would suggest its removal by an operation. The owner, however, did not agree to this, and asked me whether I could not do anything else. Therefore, I decided to make intraparenchymatous injections.

At that time I read an article, published by Dr. Ruhemann,[1]

1 "Therapeutische Monatshefte," pages 117, 158 and 420. Edited by Professor Dr. Liebreich, Berlin.

discussing the effect of iodic acid (acidum iodicum) when applied by intraparenchymatous injections.

As the following chemical formula shows, a direct separation of free iodine takes place (status nascendi):

$$IO_3H + 5\ IH = 6\ I + 3\ H_2O.$$

The iodic acid becomes reduced while in the organism. This can be proved by the presence of iodide of potash in the urine.

It has been proved by several medical men that the human organism can stand a great deal of iodic acid and its salts without disagreeable consequences. It might be added that Dr. Ruhemann used the soda salt of iodic acid with good success in chronic rhinitis and pharyngitis.

According to Dr. Ruhemann's experience I used the following prescription for the injections:

| | | |
|---|---|---|
| ℞.—Acid-iodic | . . . . . . . | 2.0 |
| Papayotin | . . , . . . . | 1.0 |
| Aq. Dist. | . . . . . . . | 20.0 |
| M. S.—Solution. | | |

I made injections of this solution every other day for eight days; thereupon I continued the injections for sixteen days longer, but only one injection every four days. The mammary gland and the syringe were disinfected thoroughly before every application. The injections of iodic acid[1] seemed to be rather painful, and the last ones much more than the first. I commenced the injections at the anterior part of the tumor, gradually going to the posterior end. Shortly after each injection a large infiltration of the surrounding tissue took place, but had almost disappeared when the time for the following injection arrived. Six weeks after the treatment was stopped the tumor had shrunk wonderfully, being only as large as a hen's egg, which size the tumor has kept since.

Internal treatment consisted of administering five grammes of iodide of potash every day for six weeks.

[1] Dr. Ruhemann used the parenchymatous injections of iodic acid in treating struma, hygroma, cystoma, and hydrocele. The dose was 1–2 c.cm. per injection. The dose of the first four injections was 1 c.cm. per dose. The dose of the last four injections was 2 c.cm. per dose.

